# LINC01589 serves as a potential tumor-suppressor and immune-related biomarker in endometrial cancer: A review

**DOI:** 10.1097/MD.0000000000033536

**Published:** 2023-04-14

**Authors:** Ruixin Chen, Jian An, Yan Wang, Lingling Yang, Qingping Lin, Yanlong Wang

**Affiliations:** a Department of Gynecology, Women and Children's Hospital, School of Medicine, Xiamen University, Xiamen, China.

**Keywords:** immune cells, LINC01589, predictive value, prognosis, uterine corpus endometrial carcinoma

## Abstract

Currently, increasing attention is being paid to biomarkers in endometrial cancer. Immune infiltration of the tumor microenvironment has been shown to significantly affect the overall survival (OS) of uterine corpus endometrial carcinoma (UCEC) patients. LINC01589 is a long non-coding RNA (lncRNA) that is rarely reported in cancer and is assumed to play a role in immune regulation. We therefore evaluated the role of LINC01589 in UCEC using the Cancer Genome Atlas (TCGA) database. We analyzed the expression of LINC01589 using the gene expression profiles of LINC01589 in the UCEC projects in TCGA. Comparisons between the differentially expressed genes (DEGs) of the cancer and adjacent normal tissues of the UCEC projects revealed that LINC01589 expression was decreased in UCEC tissues. A multivariate cox regression analysis indicated that LINC01589 upregulation could serve as an independent prognostic factor for survival. Furthermore, there was a positive correlation between LINC01589 expression and B cell, T cell, NK cell, monocytic lineage, and myeloid dendritic cell infiltration in UCEC patients. In addition, 5 clusters of hub genes were detected by comparison of different expression levels of LINC01589 in the UCEC groups. The analysis of the reactome pathway using gene set enrichment analysis (GSEA) revealed immune-related pathways, including CD22-mediated B cell receptor (BCR) regulation and antigen-activated BCRs, leading to the generation of second messengers and complement cascade pathways that were significantly enriched in the high LINC01589 expression group. Thus, LINC01589 may serve as a prognostic biomarker, as it is associated with immune infiltration in UCEC.

## 1. Introduction

Uterine corpus endometrial carcinoma (UCEC) is one of the most common gynecological malignant tumors that plague women health.^[1,2]^ UCEC develops relatively slowly, but the growth and rate of metastasis poses a challenge to its treatment.^[3]^ Encouragingly, increased screening for UCEC has resulted in a better prognosis for patients.^[2,4]^ Furthermore, analyzing the alterations in the tumor micro-environment allows clinicians to devise more efficient treatments and accurately predict patient survival, as well as predict disease-free survival status by combining the clinical indicators and characteristics of UCEC patients, thus prospectively improving their quality of life.^[1,5,6]^

UCEC most commonly occurs in elderly women; however, frustratingly, there is an emerging trend of UCEC occurring in younger women.^[5,7]^ Surgery combined with adjuvant treatments is the most common course of treatment for late stage UCEC.^[8]^ For the various symptoms of UCEC, new treatment strategies are emerging.^[9,10]^ At present, immunotherapy is the most popular due to its focus on targeting and mobilizing nonspecific immune cells, including NK cells and monocyte and macrophage lineages, resulting in cytotoxic functions in cancer tissues. This is especially important during postoperative adjuvant treatment as it reduces the risk of tumor recurrence.^[11,12]^ In recent years, role of long noncoding RNAs (lncRNA) in the regulation of immune response have been revealed. Also, some studies have shown that prevention strategies targeting long non-coding RNA-related proteins are extremely promising for UCEC treatments.^[13,14]^

Previous studies have found that LINC01589 is a lncRNA that is generally >200 nt in length, and lncRNA may play a role in regulating other genes and is assumed to play a role in immune regulation.^[15,16]^ So far, there are researchers who published a report regarding LINC01589 in pancreatic cancer, in which LINC01589 was demonstrated to be involved in several pathways such as cytokine-cytokine receptor interaction and primary immunodeficiency.^[17]^ This has aroused our interest in the possible involvement of LINC01589 in the immune microenvironment regulation of UCEC tumors, especially in the activation of monocytes, complement release, antibody secretion from B lymphocytes, as well as other biological processes (BP). In addition, LINC01589 has barely been evaluated in the field of gynecologic oncology limiting the information regarding its involvement in cancer prognosis. Therefore, we analyzed the expression of LINC01589 in UCEC using the Cancer Genome Atlas (TCGA) to identify the differentially expressed genes (DEGs) associated with different expression levels of LINC01589 and tested the relationship between LINC01589 and the degree of immune cell infiltration in the tumor microenvironment. In addition, we used univariate and multivariate cox regression and subgroup analyses to analyze the prognosis of LINC01589 levels in endometrial carcinoma as well as the relationship between LINC01589 expression and different clinical characteristics. Finally, the possible mechanism by which LINC01589 is involved in tumor immune interactions was revealed.

## 2. Materials and methods

### 2.1. Data acquisition and analysis

We downloaded RNAseq data in level 3 HTSEQ-FPKM format from the UCEC project in TCGA and abandoned any RNAseq data without clinical information. Thus, a total of 543 case data with clinical information were included in this study. For later comparisons, the RNAseq transcripts in level 3 HTSEQ-FPKM format were converted into transcripts per million reads. The clinical characteristics included in this study were age, body mass index, tumor invasion ability, TP53 mutation, menopause and complications, tumor histopathological classification, tumor tissue grade, clinical stage, residual tumor status, surgical method, hormone therapy, radiotherapy as well as other characteristics, which are all recorded in Table 1. This research data report fully conforms to TCGA Publication Requirements.

**Table 1:** Characteristics of endometrial cancer patients from TCGA database.

Characters	Level	Total (n = 543)
Age (yr)		64 (14)
Tumor invasion (%)		41 (47)
Clinical stage (%)	Stage I	339 (62.4%)
	Stage II	51 (9.4%)
	Stage III	124 (22.8%)
	Stage IV	29 (5.3%)
Histologic grade (%)	G1	98 (18.4%)
	G2	120 (22.6%)
	G3	314 (59.0%)
Residual tumor (%)	R0	372 (90.7%)
	R1	22 (5.4%)
	R2	16 (3.9%)
Primary therapy outcome (%)	CR	436 (92.0%)
	PD	20 (4.2%)
	PR	12 (2.5%)
	SD	6 (1.3%)
Histological type (%)	Endometrioid	407 (75.0%)
	Mixed	22 (4.1%)
	Serous	114 (21.0%)
Diabetes (%)	No	321 (72.6%)
	Yes	121 (27.4%)
Menopause status (%)	Peri	17 (3.4%)
	Post	445 (89.5%)
	Pre	35 (7.0%)
Race (%)	Asian	20 (4.0%)
	Black or African American	106 (21.3%)
	White	372 (74.7%)
Surgical approach (%)	Minimally invasive	201 (38.6%)
	Open	320 (61.4%)
Hormones therapy (%)	No	294 (86.7%)
	Yes	45 (13.3%)
Radiation therapy (%)	No	274 (52.9%)
	Yes	244 (47.1%)
TP53 status (%)	Mut	189 (35.9%)
	WT	337 (64.1%)

TCGA = the Cancer Genome Atlas.

### 2.2. Differential gene expression analysis

In TCGA UCEC project, the LINC01589 data were divided into 2 groups according to the median expression value of LINC01589, the LINC01589-high expression group and the LINC01589-low expression group, and the DEGs were analyzed using the DESeq2 package against the HTSEQ-Counts data with the thresholds set as follows: |log-fold change| > 1.0 and adjusted *P* value < .05.^[18]^ Heatmap and volcano plots were used to show the expression of the DEGs.

### 2.3. Gene ontology (GO) and Kyoto encyclopedia of genes and genomes (KEGG) analysis

Functional enrichment analysis of the DEGs between the LINC01589-high and LINC01589-low groups was performed according to the clusterProfiler package.^[19]^ Several GO terms were found (containing BP terms, cellular components terms and molecular function terms), and the KEGG pathways enriched in the DEGs with *P* values adjusted by the Benjamini and Hochberg method were also revealed.

### 2.4. Gene set enrichment analysis (GSEA)

To elucidate the aberrantly expressed genes between the LINC01589-high and LINC01589-low groups, a function and pathway analysis was also performed via GSEA using the clusterProfiler package.^[19]^ Pathways with an adjusted *P* value < .05 were considered as significant. The most statistically significant terms were considered as significantly enriched and these were considered to be the functional pathways in the LINC01589-high and LINC01589-low patients with UCEC.

### 2.5. Analysis of immune infiltration and its correlation with LINC01589 expression

Single-sample GSEA was performed using the GSEA package in R to compare immunocyte signatures and LINC01589 expression levels. The immunocyte signatures were considered predictors of the abundance of 24 immune cells in the UCEC samples.^[18]^ Furthermore, the correlation between LINC01589 and the infiltration of the different types of immune cells was analyzed using the Spearman correlation test. We used the Wilcoxon rank sum test to detect the association between the infiltration of immune cells and the different LINC01589 expression groups.

### 2.6. Protein–protein interaction (PPI) network construction

To collect, score, and integrate the DEGs between the different LINC01589 expression groups, we searched the STRING database to explore the PPI of these DEGs.^[20]^ We conducted a PPI network analysis with a combined score > 40% to explore the interaction among the DEGs and then applied them to the Cytoscape 3.8.0 with the MCODE plugin for detection of the potential gene clusters. MCODE scores > 3, node density cutoff = 0.1, degree cutoff and K-core = 2, node score cutoff = 0.2, and a maximum depth of 100 were used as the benchmarks for the gene module selection. In this study, the MCODE plugin was used to further determine the clusters of LINC01589 co-expression genes and neighboring genes.^[21,22]^

### 2.7. Survival analysis

Univariate and multivariate Cox analyses were used to evaluate the effect of LINC01589 expression on other clinical parameters in the development of UCEC. Our survival curve was generated using the survminer package base by a log-rank test that uses the Kaplan–Meier method.^[23]^ Nomograms were used to display the prognostic prediction model, and the model included Clinical stage, Primary therapy outcome, Radiation therapy and LINC01589 expression. A calibration curve was generated to verify the effectiveness of this model. The prognostic value of the overall survival (OS) related to LINC01589 was calculated in a forest map. Furthermore, in each subgroup of TCGA UCEC project, a subgroup survival curve was generated to evaluate the prognostic value of LINC01589 in the subgroups with different clinical characteristics.

### 2.8. Statistical analysis

For statistical analysis, the data acquired from TCGA database were merged and analyzed via R 4.0.2. Numerical and categorical variables were expressed as median (interquartile) and frequency (percentage), respectively. With regards to the relationship between LINC01589 expression and the development of UCEC, we used the t test if the data was in the norm; otherwise, we used the Kruskal–Wallis rank sum test or Wilcoxon rank sum test. The correlation between the clinical characteristic of the patients and their LINC01589 expression levels was analyzed in logistic regression. We performed ROC diagnostic experiments and obtained the area under the curve to determine the efficacy of the different LINC01589 expression levels to distinguish cancer from normal tissue. A probability value of (*P*) < .05 was considered to be statistically significant in this study.

## 3. Results

### 3.1. Aberrant expression of LINC01589 in patients with UCEC

**Figure 1.**
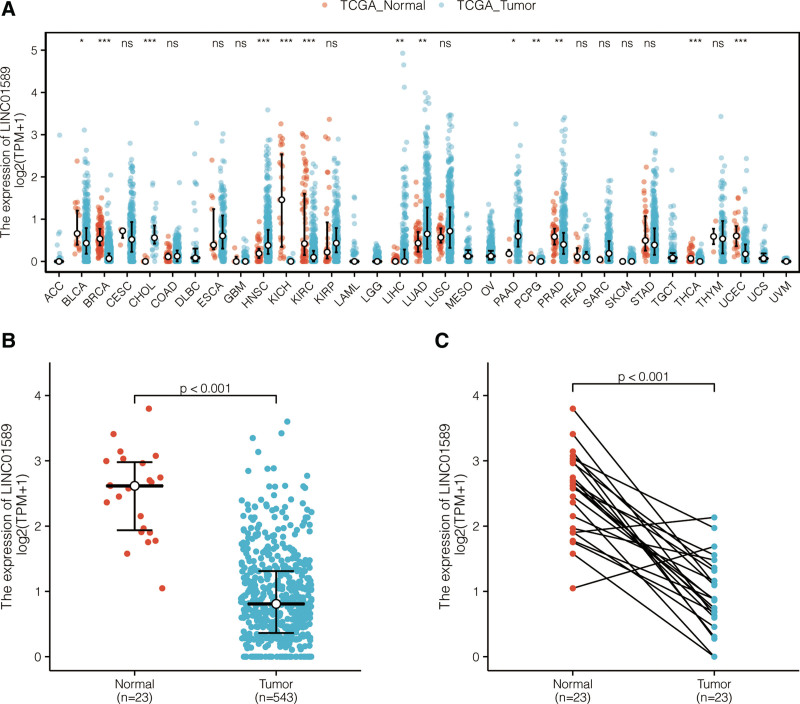
LINC01589 expression was reduced in different cancers. (A) LINC01589 expression levels in different tumors in TCGA cohorts. (B) LINC01589 expression levels in UCEC patients and normal patients. (C) LINC01589 expression levels in tissues from UCEC patients and matched adjacent normal tissues. TCGA = the Cancer Genome Atlas, UCEC = uterine corpus endometrial carcinoma.

LINC01589 data were retrieved from TCGA database to investigate its expression levels among different cancers. We further explored LINC01589 at the transcriptional level in UCEC and normal uterine tissues. These results are presented in Figure 1. Based on the data from TCGA, LINC01589 expression was found to be significantly lower in various cancer patients including UCEC patients (Fig. 1A and B). It was also revealed that the expression levels of LINC01589 were significantly lower in the paired adjacent normal tissues and UCEC tissues (Fig. 1C).

### 3.2. Prognostic value of LINC01589 in patients with UCEC

**Figure 2.**
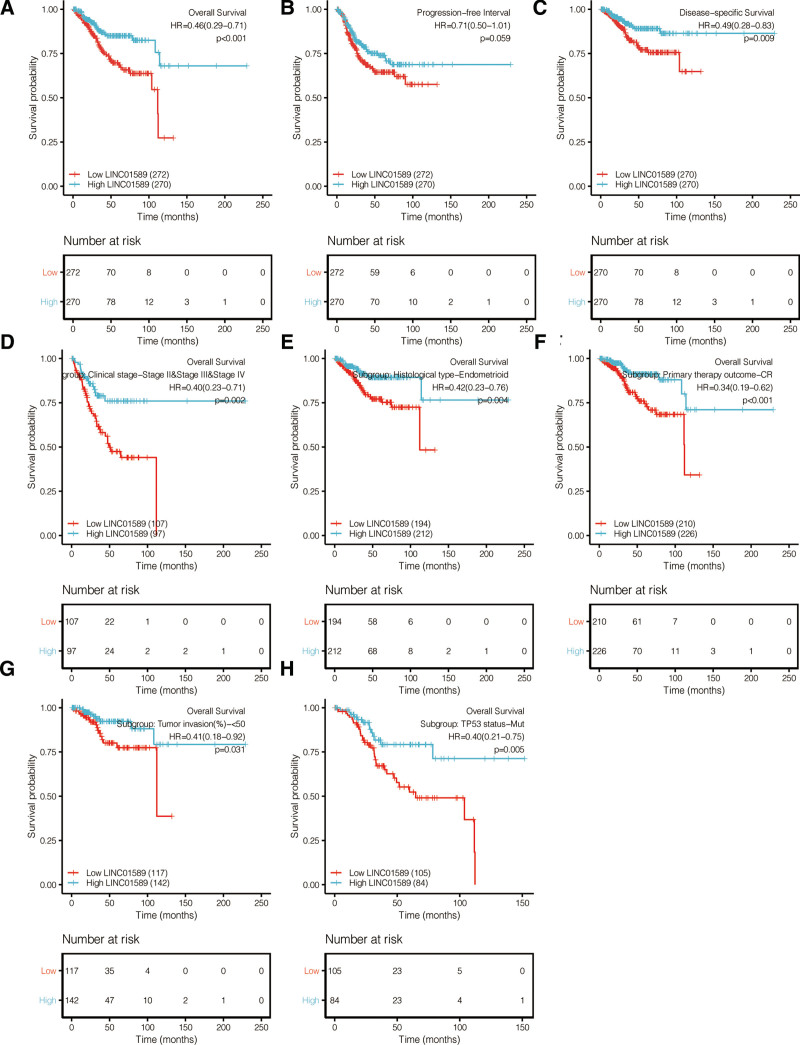
LINC01589 correlates with survival outcome. (A) Overall survival examined with respect to the different LINC01589 expressions. (B) Progression-free interval examined with respect to the different LINC01589 expressions. (C) Disease-specific survival examined with respect to the different LINC01589 expressions. (D–H) Survival analysis performed on the subgroups of patients in the UCEC projects cohorts; the stage II to IV groups (D), endometroid-type group (E), primary therapy outcome-complete recovery group (CR) (F), lower tumor invasion group (G), and TP53 mutation group (H). UCEC = uterine corpus endometrial carcinoma.

Since a negative relation of the LINC01589 expression between the normal and UCEC patient tissues was revealed, we explored the prognostic value of LINC01589 in UCEC patients. It was shown that the patients with high LINC01589 expression had a better prognosis than those with low LINC01589 expression, and that LINC01589 is an accurate preeminent signature for predicting the outcome of UCEC patients (Fig. 2A). Therefore, log-rank analysis was performed for evaluating the progression-free interval and disease-specific survival in UCEC patients. In the progression-free interval, it showed that high LINC01589-expressing patients had a better survival rate (hazard ratio, HR = 0.71, 95% CI: 0.50–1.01) (Fig. 2B). Similarly, the survival probability was much higher in the disease-specific survival in UCEC patients with high LINC01589 expression (HR = 0.49, 95% CI: 0.28–0.83) (Fig. 2C). Based on the different subgroups of clinical patients, it was shown that high LINC01589 expression correlated with a longer OS in stage II to IV groups (HR = 0.40, 95% CI [0.23–0.71], *P* = .002) as well as in the endometroid type of UCEC groups (HR = 0.42, 95% CI [0.23–0.76], *P* = .004), complete recovery groups (HR = 0.34, 95% CI [0.19–0.62], *P* < .001), in the lower tumor invasion groups (HR = 0.41, 95% CI [0.18–0.92], *P* = .031), and the TP53 mutation groups (HR = 0.40, 95% CI [0.21–0.75], *P* = .005). Thus, LINC01589 expression was an excellent biomarker associated with a positive OS outcome in UCEC patients (Fig. 2D–H).

### 3.3. Co-expression and PPI network analysis of the different expression levels of LINC01589 in UCEC

**Figure 3.**
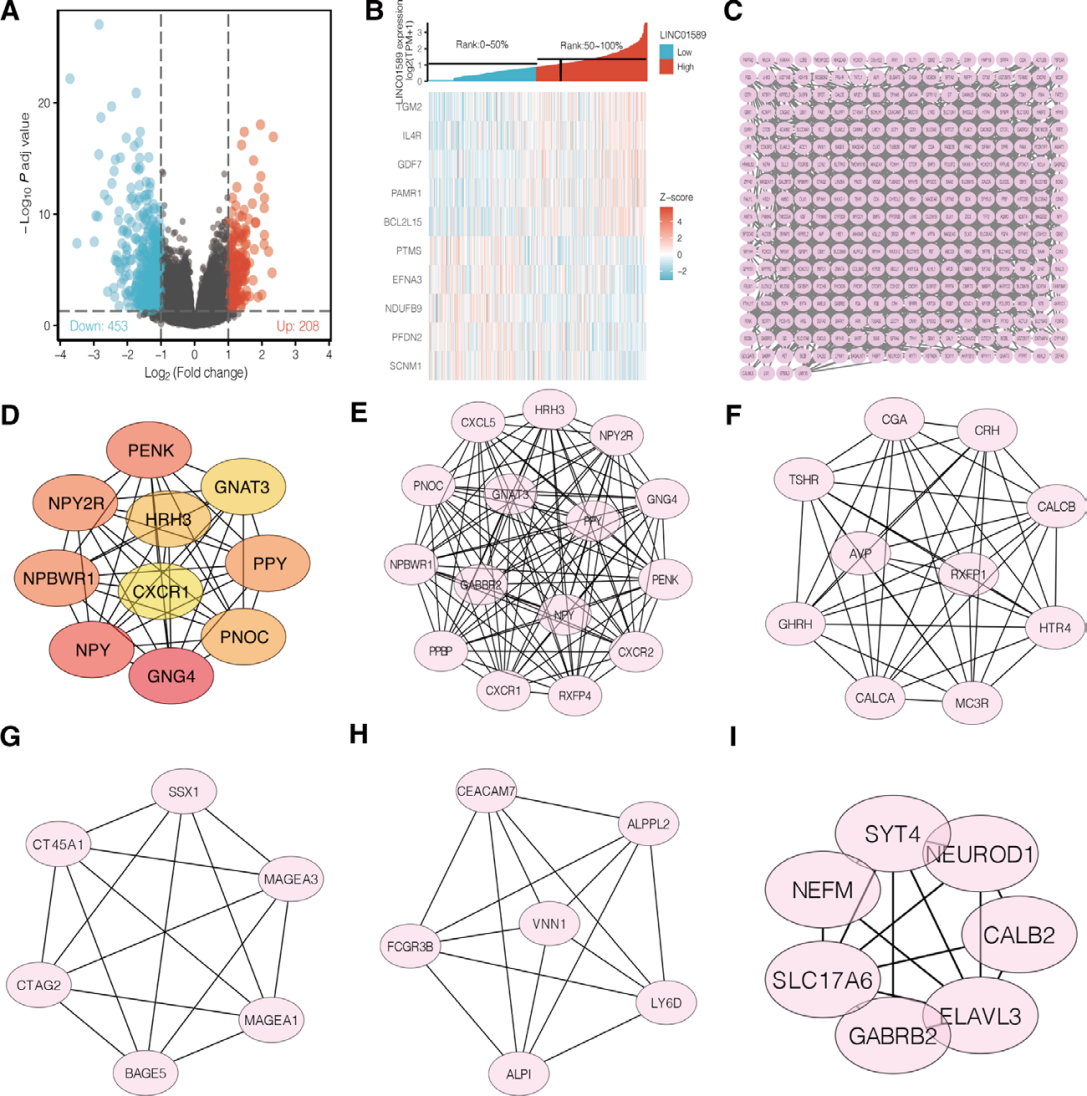
LINC01589 co-expression gene profiles in UCEC. (A) Volcano map of the different genes corresponding to the different expression levels of LINC01589. (B) Heatmap of the genes with the most obvious changes among the different expression levels of LINC01589. (C) LINC01589 co-occurrence profiles in the UCEC projects according to String database. (D) Top 10 genes with high connectivity in the different expression levels of LINC01589. (E–I) Different clusters of hub genes in the different LINC01589 expression groups. UCEC = uterine corpus endometrial carcinoma.

The correlation between the DEGs and the different transcriptional levels of LINC01589 were assessed to determine the pathological processes and pathogenic mechanisms in UCEC patients (Fig. 3A). As is shown, several genes were found to have a significant positive correlation with LINC01589 expression, including transglutaminase 2 (TGM2, *R* = 0.336, *P* < .001), IL4 receptor (IL4R, *R* = 0.329, *P* < .001), GDF7 (*R* = 0.322, *P* < .001), PAMR1 (*R* = 0.313, *P* < .001), and BCL2L15 (*R* = 0.307, *P* < .001), whereas other genes including PTMS (*r* = −0.222, *P* < .001), EFNA3 (*R* = 0.224, *P* < .001), NDUFB9 (*r* = −0.226, *P* < .001), PFDN2 (*r* = −0.234, *P* < .001), and SCNM1 (*r* = −0.235, *P* < .001) exhibited a negative correlation with LINC01589 expression (Fig. 3B). We used the DEGs related to LINC01589 that may play a role in inflammation and cancers to construct a PPI network with the STRING tool for predicting the potential protein interactions among the DEGs. In the PPI network, 328 nodes and 1142 edges were detected (Fig. 3C). To identify potential genes in the network, the MCODE plugin was used for analyzing the connectivity between the DEGs. It was elucidated that G protein gamma-4 subunit/guanine nucleotide-binding protein-4 (GNG4) was the most eligible gene with a connectivity degree = 31, followed by other hub genes, including neuropeptide Y (NPY, degree = 29), PENK (degree = 28), NPBWR1 (degree = 18), NPY2R (degree = 18), PPY (degree = 25), PNOC (degree = 16), HRH3 (degree = 16), GNAT3 (degree = 18), and CXCR1 (degree = 17) (Fig. 3D). Five key modules clusters were identified from the whole network (Fig. 3E–I).

### 3.4. Functional enrichment analysis of neighbor genes among the different expression levels of LINC01589 in UCEC

**Figure 4.**
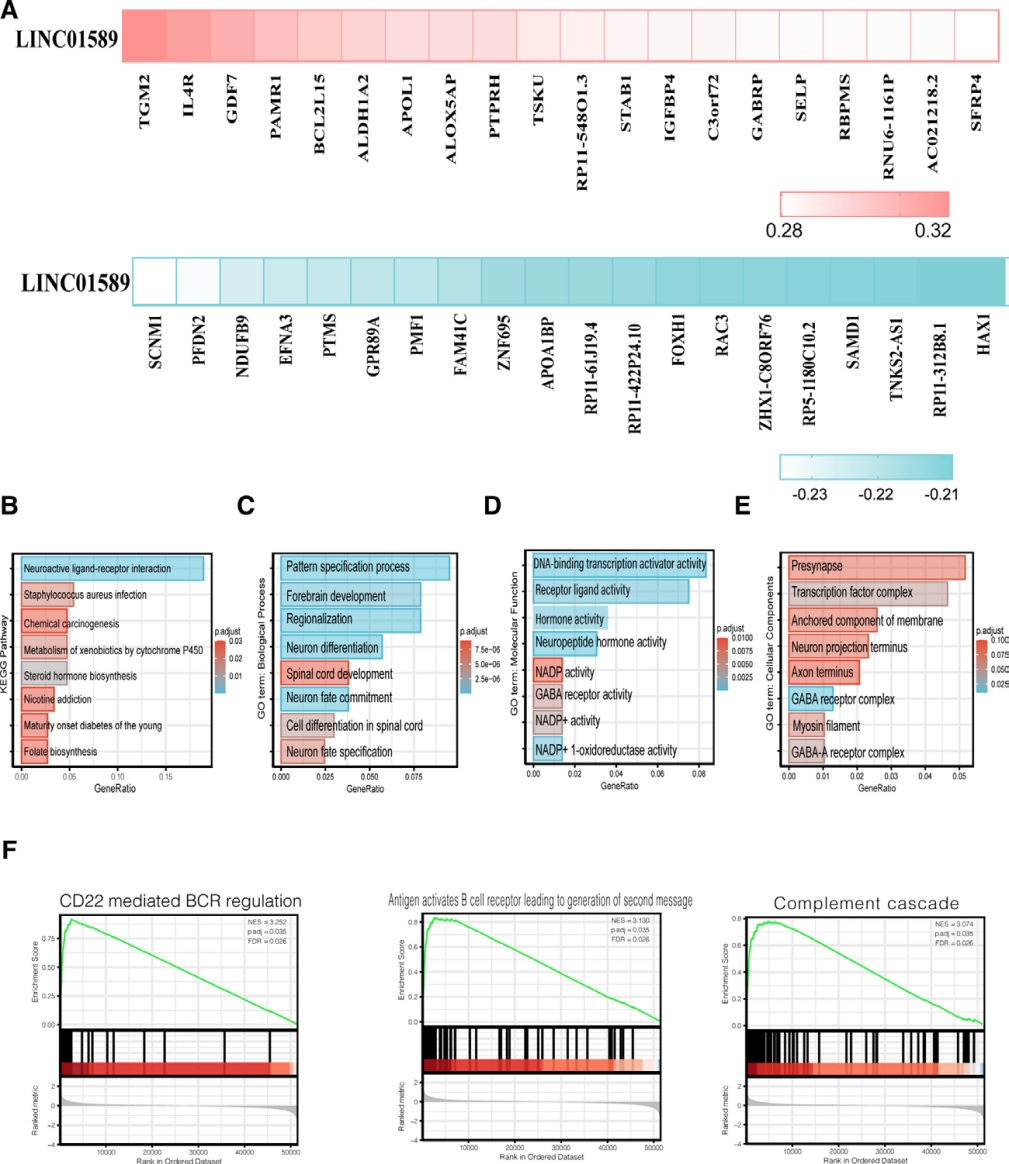
Enrichment analysis of LINC01589 co-expression genes in UCEC. (A) Heatmaps exhibiting top 20 genes that positively and negatively correlate with LINC01589 in UCEC. (B) KEGG pathway analysis of the significant LINC01589 co-occurrence genes. (C) Biological process terms of significant LINC01589 co-occurrence genes. (D) Cellular component terms of the significant LINC01589 co-occurrence genes. (E) Molecular function terms of the significant LINC01589 co-occurrence genes. (F) GSEA analysis of enriched reactome pathways of the significant LINC01589 co-occurrence genes. GSEA = gene set enrichment analysis, UCEC = uterine corpus endometrial carcinoma.

Through Pearson analysis, genes exhibiting a strong correlation with LINC01589 expression were screened out of TCGA datasets (Fig. 4A). Gene enrichment analysis was performed in R with the clusterProfiler package, which was executed to analyze the GO terms and KEGG pathways. According to the KEGG results, we found that these genes were enriched in neuroactive ligand-receptor interactions, steroid hormone biosynthesis pathways, *Staphylococcus aureus* infection, metabolism of xenobiotics by cytochrome P450, nicotine addiction, and chemical carcinogenesis, which indicated that they may be involved in different BP (Fig. 4B). Furthermore, according to the GO results, these genes were significantly enriched in pattern specification processes, neuron fate commitment, central nervous system neuron differentiation, forebrain development, cell differentiation in spinal cord, and regionalization in the BP category (Fig. 4C). In the molecular function group, they were primarily enriched in neuropeptide hormone activity, RNA polymerase II-specific DNA-binding transcription activator activity, hormone activity, receptor ligand activity, alcohol dehydrogenase (NADP+) activity, and NADP+ 1-oxidoreductase activity (Fig. 4D). The DEGs were primarily enriched in the GABA receptor complex, transcription factor complex, GABA-A receptor complex, myosin filament, neuron projection terminus, and presynapse in the cellular components category (Fig. 4E).

We then analyzed the association between the expression level of LINC01589 using GSEA and found that the correlated genes in the high LINC01589 group were enriched in CD22-mediated B cell receptor (BCR) regulation and antigen-activated BCRs, which lead to the generation of second messengers and complement cascade pathways (Fig. 4F).

### 3.5. Immune cell infiltration correlated with high LINC01589 expression in UCEC

**Figure 5.**
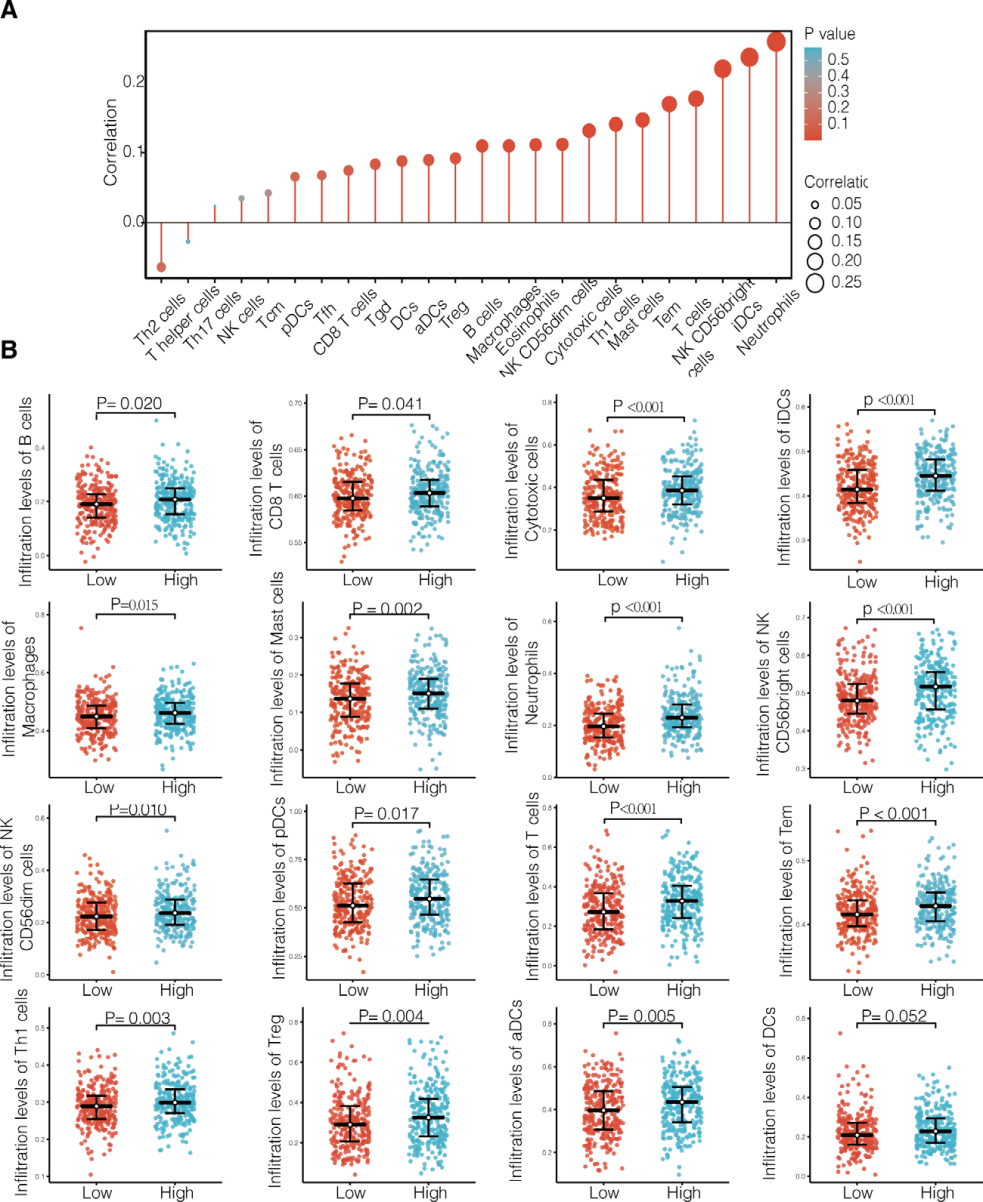
Infiltration of different immune cells between the different LINC01589 expression groups. (A) Correlation between 24 immune cells and LINC01589 expression levels. (B) The proportion of immune cells in the low and high LINC01589 groups in the UCEC cohorts from TCGA. aDCs = activated DCs, DCs = dendritic cells, iDCs = immature DCs, Tcm = T central memory cells, Tem = T effector memory cells, Tfh = T follicular helper cells, TCGA = the Cancer Genome Atlas, Tgd = T gamma delta cells, Th = helper T cells, Treg = Regulatory T cells, UCEC = uterine corpus endometrial carcinoma.

To further explore the role of LINC01589 in anti-tumor immune processes, we elucidated its correlation between 24 immune cells (Fig. 5A). We found that LINC01589 expression positively correlated with 16 types of immune cells, including B cells (*R* = 0.109, *P* = .011), cytotoxic T cells (*R* = 0.131, *P* = .002), dendritic cells (DCs) (*R* = 0.088, *P* = .041), eosinophils (*R* = 0.111, *P* = .010), immature DCs (iDCs, *R* = 0.236, *P* < .001), activated DCs (aDCs, *R* = 0.089, *P* = .037), macrophage cells (*R* = 0.110, *P* = .011), mast cells (*R* = 0.147, *P* < .001), neutrophils (*R* = 0.259, *P* < .001), CD56bright NK cells (*R* = 0.220, *P* < .001), CD56dim NK cells (*R* = 0.112, *P* = .009), T cells (*R* = 0.177, *P* < .001), T effector memory cells (*R* = 0.169, *P* < .001), Th1 cells (*R* = 0.141, *P* = .001), and Treg cells (*R* = 0.092, *P* = .032). The high LINC01589 expression group had much higher levels of B cells, T cells, NK cells, monocytic lineage, and myeloid dendritic cells (Fig. 5B).

### 3.6. Evaluating the predictive value of LINC01589 among different clinical characteristic groups in UCEC

**Figure 6.**
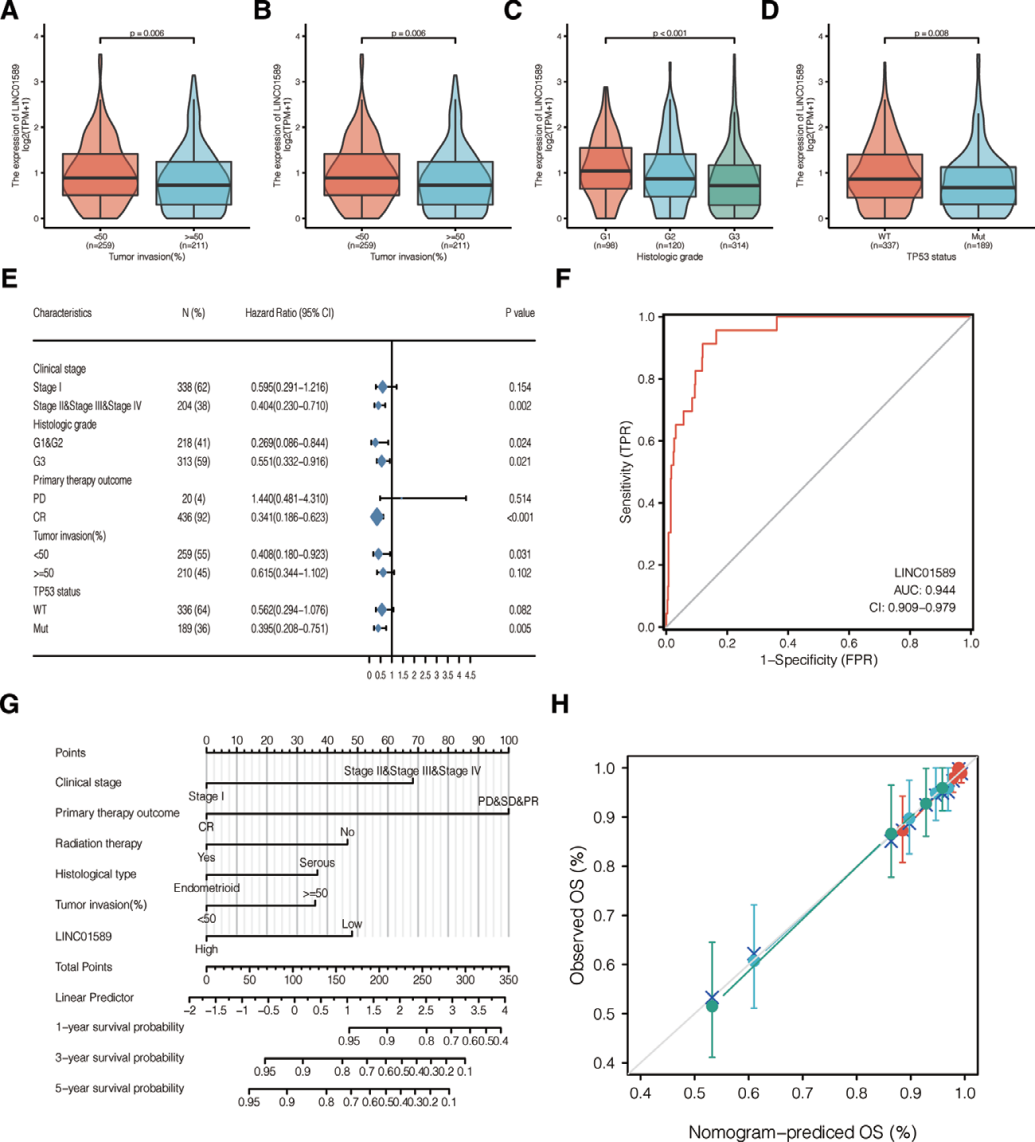
LINC01589 expression in subgroups of patients with UCEC as well as diagnostic and prognostic value of LINC01589. (A–D) Expression level of LINC01589 in patients with different ages (A), tumor invasion (B), histological grades (C), and TP53 status (D). (E) The prognostic value of LINC01589 in various subgroups of endometrial carcinoma patients (UCEC). (F) Receiver operating curve (ROC) curves according to the LINC01589 expression in TCGA. (G) Nomogram models were used to evaluate the impact of LINC01589 expression and the different clinical characteristics on overall survival in the UCEC cohorts from TCGA. (H) Calibration curve including clinical stage, primary therapy outcome, radiation therapy, tumor invasion, histological type, and LINC01589 expression. TCGA = the Cancer Genome Atlas, UCEC = uterine corpus endometrial carcinoma.

We assessed the expression levels of LINC01589 among the 543 cases of UCEC patients with different clinical characteristics. The transcriptional levels of LINC01589 were significantly lower in UCEC patients who were elderly (age > 60 years, *P* = .007) (Fig. 6A) and in the higher tumor invasion (>50%) groups (*P* = .006) (Fig. 6B). In UCEC patients with a high histologic grade (especially in G3 patients) (*P* < .001), LINC01589 was found to be significantly reduced (Fig. 6C). In addition, LINC01589 expression was also lower in patients with the TP53 mutation (*P* = .008) (Fig. 6D).

The univariate proportional hazard model was used for analyzing the key clinical parameters as well as the TP53 mutation status. In TCGA datasets, multivariate cox analysis demonstrated that LINC01589 was an independent prognostic biomarker for UCEC patients (Table 2). And the prognostic value of LINC01589 in various subgroups of endometrial carcinoma patients is presented in Figure 6E. Furthermore, LINC01589 could serve as a predictive indicator of UCEC (area under the curve = 0.944) (Fig. 6F). According to multivariate hazard analysis, a nomogram was constructed for predicting the 5-year OS based on the variable of clinical stages, LINC01589 expression level, percentage of tumor invasion, histological type of UCEC, receipt of radiation therapy, and primary therapy outcome. The C-index of the nomogram for predicting OS was 0.818 (0.787–0.848) (Fig. 6G). The nomogram-predicted OS showed an excellent correlation with the observed OS values (Fig. 6H).

**Table 2:** Cox regression analysis between overall survival outcome and clinical characteristic of UCEC patients.

Characteristics	Total (N)	Univariate analysis		Multivariate analysis	
		HR (95% CI)	*P* value	HR (95% CI)	*P* value
Clinical stage (Stage II&Stage III&Stage IV vs Stage I)	542	3.667 (2.377–5.657)	<.001	2.695 (1.205–6.027)	.016
Histologic grade (G3 vs G1 & G2)	531	3.227 (1.872–5.563)	<.001	1.446 (0.602–3.471)	.409
Residual tumor (R1 & R2 vs R0)	409	3.261 (1.852–5.744)	<.001	1.708 (0.752–3.877)	.201
Tumor invasion (%) (≥50 vs <50)	469	2.858 (1.773–4.607)	<.001	1.483 (0.703–3.130)	.301
Primary therapy outcome (CR vs PD&SD&PR)	474	0.131 (0.079–0.218)	<.001	0.217 (0.094–0.497)	<.001
Histological type (serous vs endometrioid)	520	2.874 (1.865–4.430)	<.001	1.349 (0.543–3.351)	.519
Diabetes (yes vs no)	442	1.241 (0.771–1.997)	.374		
Menopause status (Post vs Pre & Peri)	496	1.031 (0.497–2.139)	.934		
Age (>60 vs ≤60)	540	1.807 (1.133–2.884)	.013	1.714 (0.749–3.922)	.202
Weight (>80 vs ≤80)	520	1.112 (0.727–1.701)	.623		
Height (>160 vs ≤160)	513	1.222 (0.796–1.875)	.359		
BMI (>30 vs ≤30)	511	1.047 (0.682–1.606)	.833		
Race (White vs Asian & Black or African American)	497	1.055 (0.645–1.727)	.83		
Surgical approach (minimally invasive vs open)	520	1.328 (0.862–2.047)	.198		
Hormones therapy (yes vs no)	339	0.862 (0.408–1.824)	.699		
Radiation therapy (yes vs no)	518	0.623 (0.402–0.964)	.034	0.338 (0.173–0.662)	.002
TP53 status (Mut vs WT)	525	2.264 (1.483–3.457)	<.001	0.785 (0.331–1.862)	.583
LINC01589 (High vs Low)	542	0.458 (0.295–0.711)	<.001	0.463 (0.230–0.928)	.03

BMI = body mass index, UCEC = uterine corpus endometrial carcinoma.

## 4. Discussion

In our study LINC01589 was identified as a lncRNA that could serve an important role in the suppression of UCEC progression. Increasing evidence has demonstrated the importance of lncRNA in altering the cancer immune environment.^[24,25]^ Accumulating evidence has shown a correlation between lncRNA and tumorigenesis and metastasis, demonstrating that lncRNAs can modulate tumor progression.^[26,27]^ However, the prognostic value of LINC01589 as well as its underlying mechanisms have not yet been explored.

We explored the expression of LINC01589 among different types of tumors, with further analysis into the role of LINC01589 in UCEC. It was found that high LINC01589 expression may accurately predict a better clinical outcome in UCEC patients. Moreover, we found that low expression of LINC01589 significantly correlated with a worse OS rate as well as progression-free interval and disease-specific survival. Our data demonstrated that LINC01589 is significantly downregulated in UCEC patients with a worse clinical outcome. As is shown in the subgroup analysis, high expression levels of LINC01589 predicts a better OS outcome in UCEC patients among the elderly (age > 60 years) and higher tumor invasion (>50%) groups as well as in patients with higher histology grades (especially in G3 patients).

We further explored the molecular characteristics of UCEC according to the differentially expressed LINC01589 levels. There were frequent genetic alterations in accordance with LINC01589 expression in UCEC. TGM2 catalyzes the crosslinking of proteins by an epsilon-gamma glutamyl lysine isopeptide bond.^[28,29]^ According to the correlation analysis, LINC01589 has a positive relationship with TGM2 and IL4R. Recently, TGM2 was found to correlate with chemoresistance to cisplatin through the activation of mitogen-activated protein kinase and AKT serine/threonine kinase pathways.^[30]^ Furthermore, TGM2 could also serve as a target for angiogenesis, cell cycle arrest, and early apoptosis in other cancers.^[31–34]^ Meanwhile, we turned our attention to the IL4R, which is involved in anti-tumorigenesis. IL4R is thought to be a strong regulator of metastasis, proliferation, and survival in various cancers.^[35,36]^ IL4R is also capable of regulating the glucose metabolism which promotes cancer progression.^[37]^ In contrast, we found that LINC01589 negatively correlated with PFDN2 and EFNA3. It is well known that PFDM2 is involved in sex tubulin-interacting neighbor proteins which has been shown to correlate with resistance against Texans in cancer.^[38]^ PFDN2 is also closely associated with poor prognosis in cancers and is located on q23.3, whose copy-number gain correlates with poor survival outcome.^[39]^ As for EFNA3, which is also known as a non-coding RNA, it is involved in inducing ephrin-A3 protein accumulation, which are cell surface proteins that regulate diverse BP by modulating cellular adhesion and repulsion, thus promoting cancer metastasis.^[40]^ The complexity of the genetic alterations in UCEC is worth noting, and, here, we demonstrated a correlation among the DEGs and LINC01589 groups in UCEC patients, suggesting that LINC01589 may play a role in inhibiting tumor progression in UCEC.

We then focused on the hub genes in the LINC01589 groups which could be key factors in the pathological processes of UCEC. We found that these genes are primarily related to different pathological process resulting in the suppression of tumor progression, such as GNG4 and NPY. Previous studies have elucidated that GNG4 participates in the core interaction network of colon cancer, making it a significant prognostic factor that plays a crucial role in BP correlating to suppression of tumor cell growth.^[41]^ Moreover, it was demonstrated that methylated NPY could serve as a universal liquid biopsy marker in cancer patients treated with regorafenib; as the median survival for patients with methylated ctDNA levels above the median was 4.3 months compared to 7.6 months with ctDNA levels below the median, (*P* < .001) and the median time from increasing methylated ctDNA levels to disease progression was 1.64 months (range 0.46–8.38 months), this demonstrates that higher methylated ctDNA levels indicate a poor prognosis with a relative shorter disease progression interval.^[42]^

Meanwhile, we found that these DEGs are primarily related to CD22-mediated BCR regulation and antigen-activated BCRs, leading to the generation of second messengers and complement cascade pathways, according to the GSEA. Antigen-activated BCRs generate secondary messengers, which are crucial in the regulation of inflammation and immune transduction signals.^[43–45]^ Another study demonstrated the key role of CD22-mediated BCR regulation in mediating apoptosis in cancers.^[46]^ Moreover, complement cascade pathways are notably involved in cancers according to previous studies.^[47,48]^ Furthermore, complement cascade pathways have been shown to suppress UCEC progression by regulating endometrial cell proliferation and recruitment.^[49,50]^ Thus, our results may provide evidence on the correlated pathways among the differentially expressed LINC01589 groups in UCEC.

Furthermore, we also sought to characterize the immune cells in UCEC according to the differentially expressed LINC01589 groups. Increasing evidence has demonstrated that infiltration by immune cells, especially cytotoxic T cells, dendritic cells, and macrophages, could be involved in the suppression of tumor progression.^[51,52]^ In this study, we found that the infiltration of immune cells, including B cells, macrophages, NK cells, and dendritic cells, among the differentially expressed LINC01589 groups positively correlated with LINC01589 expression levels.

Differentially expressed LINC01589 may distinguish patients with UCEC from healthy individuals. Our data indicated that LINC01589 along with clinical stages, histologic grade, residual tumor, percentage of tumor invasion primary therapy outcomes, histological types, radiation therapy, and patients age as well as TP53 status were determining factors in the prognosis of UCEC patients. In particular, according to the multivariate cox hazard model, it was indicated that UCEC patients with low LINC01589 expression levels and high clinical stages, who did not receive radiation therapy and who did not have complete response after primary therapy outcome, may have a worse OS outcome.

There are also some limitations in our study. Most notably, we showed that differentially expressed LINC01589 was associated with immune status through different pathways, specifically LINC01589 was shown to participate in immune infiltration, but this has not been verified by in vitro studies. Thus, in vitro studies will be required to validate the gene-expression profile.

## 5. Conclusions

In conclusion, our study demonstrated the prognostic value of LINC01589 in UCEC patients and provided insights into the genes that correlate with LINC01589 expression, especially the DEGs among the different LINC01589 expression groups that participate in the different BP of UCEC progression. We identified LINC01589 as a promising biomarker in determining the prognosis of UCEC patients.

These results support the hypothesis that LINC01589 has prognostic value in determining the clinical outcome of UCEC patients. Additionally, we demonstrated that the LINC01589 related-genes participate in the activation of immune-related pathways.

## Acknowledgments

This study received the support from Young talent Spark Program of Women and Children hospital, School of Medicine, Xiamen University ([2018]26 to Chen RX).

## Author contributions

**Conceptualization:** Ruixin Chen, Qingping Lin.

**Data curation:** Yan Wang, Lingling Yang.

**Formal analysis:** Ruixin Chen, Jian AN.

**Funding acquisition:** Yanlong Wang.

**Investigation:** Lingling Yang.

**Methodology:** Ruixin Chen, Jian AN, Yan Wang.

**Project administration:** Qingping Lin.

**Software:** Jian AN.

**Supervision:** Qingping Lin, Yanlong Wang.

**Validation:** Yan Wang.

**Visualization:** Jian AN.

**Writing – original draft:** Ruixin Chen, Jian AN.

**Writing – review & editing:** Qingping Lin.
